# Effects of Combined Opioids on Pain and Mood in Mammals

**DOI:** 10.1155/2012/145965

**Published:** 2012-03-21

**Authors:** Richard H. Rech, David J. Mokler, Shannon L. Briggs

**Affiliations:** ^1^Department of Pharmacology and Toxicology, Michigan State University, East Lansing, MI 48864, USA; ^2^Department of Biomedical Sciences, College of Osteopathic Medicine, University of New England, 11 Hills Beach Road, Biddeford, ME 04005, USA; ^3^Department of Environmental Quality, State of Michigan, Lansing, MI 48909-7773, USA

## Abstract

The authors review the opioid literature for evidence of increased analgesia and reduced adverse side effects by combining mu-opioid-receptor (MOR) agonists, kappa-opioid-receptor (KOR) agonists, and nonselective low-dose-opioid antagonists (LD-Ant). We tested fentanyl (MOR agonist) and spiradoline (KOR agonist), singly and combined, against somatic and visceral pain models. Combined agonists induced additive analgesia in somatic pain and synergistic analgesia in visceral pain. Other investigators report similar effects and reduced tolerance and dependence with combined MOR agonist and KOR agonist. LD-Ant added to either a MOR agonist or KOR agonist markedly enhanced analgesia of either agonist. In accordance with other place-conditioning (PC) studies, our PC investigations showed fentanyl-induced place preference (CPP) and spiradoline-induced place aversion (CPA). We reduced fentanyl CPP with a low dose of spiradoline and reduced spiradoline CPA with a low dose of fentanyl. We propose combined MOR agonist, KOR agonist, and LD-Ant to produce superior analgesia with reduced adverse side effects, particularly for visceral pain.

## 1. Introduction

 This paper supports, with scientific references, the hypothesis of a clinical utility of combinations of moderate doses of (a) a selective mu opioid receptor (MOR) agonist, (b) a selective kappa opioid agonist (KOR), and (c) ultralow doses of a nonselective opioid antagonist. The authors propose this triple opioid combination to produce a superior analgesic profile while reducing adverse and possibly lethal side effects of MOR and KOR agonists. Whereas somatic and neurogenic pain of short and long terms may be controlled with use of the proposed combination, the treatment should be most effective in allaying chronic visceral pain.

## 2. The Need for Improved Opioid Analgesic Drug Regimens

 MOR agonists such as morphine, methadone, fentanyl, hydrocodone, and oxymorphone are very effective analgesics, and about 23 million prescriptions are dispensed each year for extended-release and long-acting opioids alone, which represented about 10 percent of the opioid market in 2009 (April 19, 2011, teleconference with Janet Woodcock, M.D., Director, Center for Drug Evaluation and Research, U.S. Food and Drug Administration). The beneficial effects of the opioids are frequently compromised by development of tolerance, dependence, hyperalgesia, addiction, and respiratory, and cardiovascular toxicities, the latter two leading too often to fatal consequences (White and Irvine [1]; “The Hill”: Pecquet (4/19/11): “Healthwatch” blog reported, “As a first step, the FDA sent letters to opioid manufacturers on Tuesday requiring that they provide a plan for training and educating patients about the safe use, storage and disposal of opioids. They have 120 days to respond, setting in place a regulatory process that officials hope to have in place within 12 months. ‘We have determined that a Medication Guide Communication plan is not sufficient to mitigate the serious risks,' the letters state. ‘Your (strategy) must include tools to manage these risks.' The FDA missive was sent to producers of Dolophine (methadone); ms Contin, Kadian, Avinza, Embeda, Oramorph (morphines), Oxycontin (oxycodone); Exalco (hydromorphone); Duragesic (transdermal fentanyl); Butrans (buprenorphine); and Opana ER (oxymorphone)”; Hardman et al. [2]; Smith et al. [3]). Coop, who served for years as US Surgeon General, and his colleague MacKerell [4], urged the medical community to devise more effective and safer drug combinations of opioids. More recently, the FDA has now imposed new risk evaluation and mitigation strategy (REMS) requirements on marketers of extended release and long-acting opioids. This agency interaction thus supports the need for improvements in the way that opioid analgesics are prescribed and used.

 Smith also called for improved analgesics, indicating that there were no ideal opioid preparations [5]. He pressed for the study of combinations to enhance analgesia while reducing unwanted side effects in 6 categories: (a) to prolong analgesic duration, (b) to increase analgesic efficacy (synergy), (c) to diminish or minimize adverse side effects, (d) to reduce nonbeneficial effects, (e) to reduce tolerance and development of hyperalgesia, and (f) to decrease dependency and addiction liability. Piercefield et al. [6] cited many overdose deaths in the United States that were related to methadone and other MOR agonists, mainly among males 35–54 years of age. In addition to significant opioid abuse, lethal outcomes occur due to provider and patient unfamiliarity with proper dosing regimens to ameliorate these problems with opioid dosing. Williamson et al. [7] indicated that many preventable overdose deaths occurred with methadone use in Australia, both prescribed and illegally diverted. Indeed, globally, risk of serious medical consequences of opioid use has not decreased and there remain specific therapeutic needs for safer and more effective opioid preparations.

## 3. Initial Studies with Mixed Opioid Agonists

 The staff at Dr. Rech's Michigan State University neuropsychopharmacology research laboratory began animal studies with mixed opioid agonists in the 1980s, seeking an improved opioid analgesic agent against colorectal distension (CRD) nociception (visceral pain model) in feline subjects (Sawyer et al. [8], Sawyer & Rech [9], Sawyer et al. [10]). Feline subjects react to MOR agonists with a manic-like disoriented excitation, having dominant brain excitatory opioid receptors (Robertson and Taylor [11]). This prompted us to seek a calmer, sedating analgesic response with KOR-agonist activity. While these cats reacted to oxymorphone with agitated excitement, a different behavior was seen when they received the mixed action MOR/KOR agonist butorphanol subcutaneously (s.c.). The subjects remained quiet and even purred when petted over the first postdrug hour, with a moderate antinociceptive response that showed a ceiling effect. During the second postdrug hour, as the butorphanol antinociception waned, the cats became irritable. They flinched when touched and startled to a sharp noise. Nalbuphine and pentazocine, agonist-antagonist KOR agents, had less effective antinociception and exhibited a second-hour phase of irritation similar to that seen with butorphanol.

 Canine subjects were also tested for butorphanol antinociception in the CRD procedure (Houghton et al. [12]; Sawyer et al. [13]). This species, which possesses dominant brain inhibitory opioid receptors, was calm and sedated during the first hour after butorphanol or oxymorphone injection. During the second hour butorphanol-treated dogs reacted with irritability similar to that phase observed in the cat. Pain relief was similar to that in feline subjects, but was accompanied by a slight respiratory depression and reduced heart rate. Thus, in both species, butorphanol's MOR agonist component was evident during the first postdrug hour, whereas KOR-agonist signs of agitation emerged during the second postdrug hour.

 In a later study, Dr. Briggs et al., as a graduate student, examined the interactions of butorphanol combined with oxymorphone in the cat [14]. The combined drugs exhibited synergistic antinociception in the CRD over the response to each drug administered separately, but without the initial phase of excitement seen with oxymorphone alone.

## 4. Studies of Selective MOR and KOR Agonist Antinociception, Alone and Combined

 These experiments were performed with Dr. Briggs and supported her thesis dissertation under Dr. Rech's mentorship (Briggs, S.L.: Interactions of mu- and kappa-opioid agonists, Michigan State University, 1996). In these experiments, the selective KOR-1 agonists spiradoline and enadoline, as well as the selective MOR agonist fentanyl, were tested for antinociception in the cold-water tail-flick (CWTF) assay (Briggs et al. [15]). The CWTF assay, a somatic pain nociceptive test, was chosen since Pizziketti et al. [16] found it to be efficient and sensitive to both opioid agonists. The opioids tested in these experiments were shown to be full agonists for maximal antinociception. Both spiradoline and enadoline were as efficacious, but less potent analgesics than fentanyl. Furthermore, naloxone (NLX), a nonselective opioid antagonist, attenuated both fentanyl antinociception, at 0.1 mg/kg, and KOR-agonists antinociception, at 0.5 mg/kg. Fentanyl antinociception was markedly reduced in methadone-tolerant animals, whereas spiradoline antinociception was unchanged. Spiradoline antinociception was nullified by pretreatment with nor-binaltorphimine (n-BNI, KOR-1-specific antagonist). Fentanyl antinociception was abolished by beta-funaltrexamine (b-FNA, MOR-specific antagonist). And, as expected, b-FNA pretreatment did not alter spiradoline antinociception, nor did n-BNI pretreatment alter fentanyl antinociception.

 Fentanyl and spiradoline were also tested in rats for pain relief in the CRD procedure, a visceral pain model, along with oxymorphone and enadoline (Briggs and Rech [17]). All showed fully effective antinociception when administered separately. Combining fentanyl and spiradoline produced additive (low doses) or supra-additive (high doses) effects. The supra-additive combination was attenuated by either b-FNA or n-BNI (greater with the latter). When b-FNA and n-BNI were tested against the antinociception of single doses in CRD, paradoxical effects again occurred: the fentanyl effect was not antagonized by b-FNA, whereas the spiradoline effect was. Thus, complex paradoxical interactions took place in the CRD test, as opposed to the expected results as seen using the CWTF procedure.

 Rech combined fentanyl and spiradoline in the CWTF (see Briggs et al. [15] above), to test for an additive antinociceptive response in rats (not previously published). In this last test, respiratory depression to fentanyl alone (0.008 mg/kg) was reduced when fentanyl (0.004 mg/kg) and spiradoline (0.56 mg/kg) were combined in ED50 doses to yield comparable antinociceptive levels for agonists given singly. This result resembled those respiratory effects reported by Verborgh et al. in rats [18] and Houghton et al. in dogs [12], both of which showed reduced respiratory depression to a MOR agonist by combining it with a KOR agonist.

 An article by Negus et al. [19], which described results somewhat similar to the CRD and CWTF studies in rats by Briggs and Rech [17] and Briggs et al. [15], is reviewed here for comparison and contrast. Negus et al. tested fentanyl and U69593 (KOR-1 agonist) interactions in monkeys in three behavioral assays: (a) schedule-controlled responding for food (fixed ratio 30), (b) thermal nociception (50°C) for tail-withdrawal latencies (somatic pain model), and (c) schedule-controlled self-administration of both agonists, alone and combined. In the food assay both agents reduced rate of responding, and combined drugs produced subadditive effects. Both drugs alone induced dose-dependent antinociception, and combined drugs yielded additive antinociception. In the self-administration assay, fentanyl maintained responding for the drug, whereas U69593 did not. Combined drugs caused reduced self-administration levels with increasing fixed-ratio values. Thus, activation of both mu and kappa receptors with combined drugs appeared to reduce addiction liability while maintaining the additive decrease in pain.

 A conventional wisdom indicating that combined MOR and KOR opioids had no role in pain relief is likely to have been related to interactions with the early KOR agonist-antagonists, pentazocine, and nalbuphine. After development of selective KOR-1 agonists by The Upjohn Company, some studies that were performed by non-Upjohn researchers with U-50,488H continued to report antagonism of MOR-agonist antinociception by U-50,488H, as follows. Pan et al. [20], Pan [21], Bie and Pan [22], and Tershner et al. [23] studied microinjections of the agents into brainstem nuclei. They showed KOR agonists to antagonize MOR-agonist antinociception using a somatic pain (tail-flick) test. The same group (Meng et al., [24]) tested rats with U69593 microinjected into the brain stem-rostral-ventromedial medulla (RVM), using tail-flick latency and RVM activity. The KOR agonist was proposed to be either pronociceptive (direct effect on “OFF cells”) or antianalgesic by presynaptic and postsynaptic inhibition of glutamate inputs to RVM OFF cells.

 In 2002, McNally and Akil authored a book chapter [25] on opioid pain modulation, emphasizing that KOR agonists antagonized MOR-agonist analgesia. In contrast to that emphasis on antagonism of MOR-agonist activity by KOR agonists, there are many references (presented below) which support the utility of combined MOR- and KOR-agonists for synergistic action in the relief of pain. But prior to presentation of this listing, a review of the role of ultralow-doses of nonselective opioid antagonists is provided below. Ultralow doses of nonselective opioid antagonists, in combination with MOR and KOR agonists, are proposed here as representing a potentially superior clinical treatment to reduce pain, especially of the visceral type.

## 5. Ultralow-Dose Effects of Nonselective Opioid Antagonists

 Naloxone (NLX) and naltrexone (NTX), in doses 50 to 150 times less than those used to antagonize antinociception of MOR and KOR agonists, have induced surprising effects in experimental models. Shen and Crain found these doses of antagonists to markedly enhance mu-opioid agonists' antinociception. Tolerance, physical dependence, and opioid-induced hyperalgesia were reversed to marked analgesia, along with reduced side effects [26–30]. These paradoxical results were defined more fully by Angst and Clark in a review [31], presenting the concept of competing opioid excitatory and inhibitory receptors in mammalian nervous systems, expressing the activation of excitatory mu receptors as opioid-induced hyperalgesia (OIH).

 Tilson et al. [32] originally described hyperalgesia in rats following 3 days of s.c. morphine administration, followed by withdrawal. The morphine antinociceptive threshold in an electrical nociceptive tail-flick test was found to be reduced to 30 percent below the control (saline s.c.) nociceptive response. The authors offered the results as a measure of the intensity of morphine withdrawal. Low-dose nonselective antagonist effects on MOR excitatory opioid receptor mechanisms have been reported by many other researchers (see Christrup [33], Chu et al. [34], Field et al. [35], Powell et al. [36], Juni et al. [37], Abul-Husn et al. [38], McNaull et al. [39], and Tsai et al. [40]). Similar interactions between low-dose antagonists and KOR agonists occur, though less dramatically, in enhanced KOR-1-agonist effects on excitatory KOR opioid receptors. Examples are reports by Clemens and Mikes [41], Largent-Milnes et al. [42], Sloan and Hamann [43], and Webster et al. [44].

## 6. Other Antinociceptive Interactions of KOR Agonists in Animals

 Bhargava et al. [45] determined that KOR activation by U-50,488H did not modify the development of antinociceptive tolerance to morphine in rats. However, Bie and Pan [22], cited earlier, found KOR agonists injected into the brain stem nucleus raphé magnus to attenuate MOR-agonist antinociception (to tail-flick, somatic pain model). Withdrawal-induced hyperalgesia, presumably by inhibition of glutamate transmission, was also suppressed. Black and Trevethick [46] proposed that KOR activation was especially effective in suppressing visceral pain (also see Yaksh [47]). Disrupting the KOR gene in mice impaired KOR-agonist antinociception of visceral pain and attenuated morphine withdrawal (Simonin et al. [48]).

 U-50,488H antagonized respiratory depression of DAMGO (MOR-agonist peptide) and morphine, the effects being reversed by the antagonist n-BNI (Dosaka-Akita et al. [49]). Field et al. found enadoline (KOR-1 agonist) to reverse hyperalgesia and allodynia in a rat model of surgically induced pain [35]. The KOR agonist peptide Dynorphin A-(2–17) reduced morphine tolerance in mice (He and Lee [50]). KOR-agonist activity in rat periaqueductal gray was found to attenuate morphine tolerance and dependence (Herra'ez-Baranda et al. [51]). Jang et al. [52] used nalbuphine to block morphine tolerance and dependence in rats. Khotib et al. [53] injected U-50,488H s.c. for 7 days in mice, upregulating morphine receptor function and enhancing antinociception. Ko et al. [54] injected U-50,488H into monkeys to reduce morphine-provoked pruritis, while maintaining or enhancing the antinociception effect of morphine.

 As described in a series of articles, Sutters et al. [55], Miaskowski et al. [56, 57], and Miaskowski and Levine [58] microinjected DAMGO and U-50,488H intracerebroventricularly (i.c.v.) and intrathecally (i.t.) to test antinociceptive interactions against mechanical nociception (visceral pain). They obtained antagonistic or enhanced effects, the latter with reduced side effects of both agonists. Most combinations resulted in synergistic antinociception, the greatest with i.c.v. DAMGO and i.t. U-50,488H. Mechanisms were proposed involving multiple brain-spinal ascending and descending neuronal loops, with mu and kappa receptors at junctions of shared components. Background evidence relating to these concepts was presented by Yaksh [47] and his colleague, Schmauss [59]. They had mapped MOR and KOR receptor sites with microinjections into brain stem and spinal-dorsal-horn sites, microinjecting agonists and testing for somatic (thermal tail-flick) and visceral antinociception (writhing). These studies demonstrated that somatic and visceral pain, along with their suppression, are mediated by distinctly different pathways.

 Ren et al. [60] administered i.t. subanalgesic doses of morphine and dynorphin A (1–13) in combination, which resulted in marked antinociceptive synergy, assessed by tail-flick latency in rats. However, when dynorphin A (1–13) was injected i.c.v., the pain relief from i.c.v. morphine was markedly antagonized. Therefore, combined MOR- and KOR-agonist effects greatly depend upon sites of administration. Ross and Smith [61] and Nielsen et al. [62] determined that acute oxycodone antinociception was attenuated by pretreatment with n-BNI, and that oxycodone and morphine had distinctly different profiles of action, convincingly proving oxycodone to be a KOR agonist. With chronic use, however, oxymorphone, the major metabolite of oxycodone, accumulates, adding a MOR-type antinociception to the effects of oxycodone. In humans, however, oxycodone is metabolized to oxymorphone in too low amounts (10%) to affect pain relief (Chinalore et al. [63], Tompkins et al. [64], and Zwisler et al. [65]). However, Ross et al. [66] combined a low dose of oxycodone with morphine in rats, i.c.v., i.p, and s.c., to cause synergistic antinociception, along with reduced central nervous side effects.

 Schepers et al. [67] described results of Harley and Hammond using acute microinjections of MOR and KOR agonists into rat brain-stem rostral-ventromedial medulla (RVM) to yield a thermal antinociception that was potentiated in the presence of an inflammatory condition. Schepers' group extended those studies by injecting rats with complete Freund's adjuvant (cFA) into a paw plantar region to promote inflammation (a chronic visceral pain process). Two weeks later antinociception was induced by infusion into RVM of U69593 or DAMGO over 4 hours. Paw withdrawals were assessed by Hargreave's method. Mechanical thresholds with von Frey and Randall-Sellito methods were obtained, after which infusion of each drug produced prominent antinociception. Millan [68] tested U-50,488H and U69593 in rats subjected to noxious pressure (visceral pain), thermal and electrical stimuli. Prominent antinociception occurred to pressure, a weak response was seen to thermal stimuli, and the agonists were inactive to electrical shock (somatic pain).

 Vonvoigtlander and Lewis [69] attenuated U-50,488H antinociception in rats by pretreatment with reserpine and p-CPA (brain 5-HT depletors). Spiradoline (U-62,0676) antinociception, however, was little affected by the pretreatments. Ho and Takemori [70] determined that U-50,488H pain relief in rodents was also blocked by pretreatment with 5-HT antagonists. These results suggest that some U-50,488H effects may differ from those of spiradoline and other arylacetamide KOR-1 agonists. U-50,488H (3.2−10 mg/kg pretreatment) completely blocked development of tolerance to chronic morphine in rats (Yamamoto et al. [71]). U-50,488H (10 mg/kg) also restored antinociception in morphine-tolerant animals. Other KOR agonists (enadoline, dynorphin A-(2–17), and nalbuphine) also reversed or blocked morphine tolerance, hyperalgesia, and allodynia (see [35, 50–52] above). These results clearly support the combined treatment with chronic MOR and KOR agonists to maintain and enhance a persistent analgesia as compared to effects of chronic MOR-agonist treatment alone.

 Systemic morphine and spiradoline were compared in hot-plate, tail-flick, and acetic acid writhing in mice by Kunihara et al. [72]. Spiradoline was more potent than morphine, and tolerance developed to either agonist on chronic treatment. Spiradoline pretreatment did not inhibit the morphine antinociception in any test. Terner et al. [73] pretreated rats with an ultralow dose of NTX before injecting morphine in a thermal tail-flick paradigm. These studies demonstrated that the morphine antinociceptive response is enhanced after low-dose NTX pretreatment versus morphine control antinociceptive scores. Furthermore, they indicate that NTX reverses the development of tolerance to chronic morphine treatment.

 Sounvoravong et al. [74] compared morphine and U-50,488H for tail-pinch antinociception in a neuropathic pain model (sciatic nerve ligation). The morphine response was weak, while the U-50,488H response was similar to that in control mice. In a dynamic allodynia test, only U-50,488H produced antinociception and a decreased hyperalgesia. These findings suggest that KOR agonists are superior to MOR agonists for control of these types of pain.

## 7. Antinociceptive Interactions of KOR Agonists in Human Subjects

 Staahl et al. [75] also found that visceral pain in humans was often difficult to control with MOR agonists. They reported that oxycodone was superior to morphine for treatment of some types of visceral pain. Gear et al. [76] reported that nalbuphine increased postoperative dental pain in male patients, but not in females. Pretreatment with a subanalgesic dose of morphine reversed this response to analgesia by antagonizing this nalbuphine antianalgesic response in males.

## 8. Rewarding and Aversive Effects of Opioids and Other Drugs Reflecting Motivational (Mood) Influences

 Early studies in motivational opioid effects were aggressively pursued by Shippenberg and colleagues. Shippenberg et al. [77] tested morphine and fentanyl for development of tolerance and cross-tolerance, and interaction with U69593 tolerance, in a place conditioning (PC) procedure. Noncontingent morphine for 4 days induced tolerance to the development of conditioned place preference (CPP) on training rats in an unbiased multicompartment PC maze. Cross-tolerance to fentanyl was also established. Noncontingent injection of U69593 produced tolerance to the subsequent attempt to train subjects to the KOR agonist for conditioned place aversion (CPA). Pretreatment with non-contingent U69593 did not result in tolerance when morphine was subsequently trained for CPP, however. Shippenberg et al. [78] treated rats with complete Freund's adjuvant (cFA) for 7 days to provoke inflammation in a hind limb. Subjects were then trained for U69593 aversion (CPA), which failed to develop. Therefore, prolonged noxious inflammation by cFA interfered with the development of a CPA response to the KOR-1 agonist. These results were suggested as indicating potential clinical utility of the agonist for management of chronic pain states.

 Bals-Kubik et al. [79, 80] determined that aversive effects of MOR antagonists and KOR agonists using PC were centrally mediated. NLX (nonselective opioid antagonist) and CTOP (MOR-selective antagonist) produced CPA after s.c. or i.c.v. injections in rats. n-BNI i.c.v. did not induce CPA, but U50,488H and E-2078 (a dynorphin derivative) did. The opioids showing CPA were active in much lower doses i.c.v. than with s.c. doses. The mechanism for drug-induced aversion appears to be a blockade of brain mu responses. Shippenberg et al. [81] sought more detailed neurochemical bases for these motivational effects, thought to involve mesolimbic DA neurons. The neurotoxin 6-OHDA was microinjected bilaterally into the NAcc to abolish both morphine CPP and U69593 CPA. Lesions with 6-OHDA in some other mesolimbic nuclei did not affect the PC scores. Microinjection of the D-1 DA antagonist SCH-23390 into NAcc attenuated the PC of both agonists. A D-2 DA antagonist (sulpiride) was without effect.

 To continue their studies of aversive opioid mechanisms, Shippenberg and Bals-Kubik [82] microinjected NLX or CTOP into either the ventral tegmental area (VTA) or NAcc of rats to induce CPA. Lesions of NAcc with 6-OHDA nullified the aversion from intra-VTA CTOP, without modifying aversions from intra-NAcc CTOP or systemic NLX. The authors submitted that aversive effects caused by systemically administered opioid receptor antagonists do not depend upon mesolimbic DA neurons. Compulsive drug use, even after prolonged abstinence, involves 80–90% relapse rates (Shippenberg et al. [83]). This suggests that repeated drug use induces long-term alterations involving reactions of brain motivational systems to support the compulsion. Brain KOR functions, interacting with central MOR sites, play an essential role in driving opposing mood states. Central neurochemical changes with repeated drug use underscore vulnerabilities for addiction to opioids, cocaine and amphetamines, and alcohol, as well as to their combinations. Potential drug therapies targeting these altered systems are suggested treatment for these addictions.

 Pfeiffer et al. [84] indicated that KOR agonists are free of the undesirable side effects of MOR agonists, including euphoria. Dysphoric actions to KOR agonists were thought to be mediated via sigma/phencyclidine receptors. However, the benzomorphan KOR agonist MR 2033 was inactive at sigma/phencyclidine receptors. They studied MR 2033 in human males, finding that the drug elicited dose-dependent dysphoria and psychotomimetic effects that were antagonized by NLX. Thus, MR 2033 appears to exert these aversive effects by way of kappa receptors, implying the existence of opposing MOR/KOR motivational systems in mammalian brains.

 Another article by Shippenberg's group is that by Acri et al. [85]. Along with a host of other investigators, they studied interactions between cocaine and KOR agonists. U69593, in repeated doses, was described as downregulating pre- and postsynaptic DA D-2 receptors in rat brain striatum. This effect led to the prevention of cocaine-induced behavioral sensitization, which may have clinical relevance for the treatment of cocaine addiction.

 Olmstead and Burns [86] used PC to test the hypothesis that ultralow doses of NTX coadministered with MOR or KOR agonists would alter their rewarding or withdrawal-induced aversive effects. NTX doses (0.03–30 ng/kg) were tested against oxycodone CPP in female rats (more sensitive than males for PC). NTX, 5 ng/kg, blocked CPP of morphine, 5 mg/kg, as well as the CPA to withdrawal from chronic morphine, 5 mg/kg for 7 days. Coadministering NTX, 20 pg/kg, also blocked the CPA to withdrawal from chronic oxycodone (KOR agonist), 3 mg/kg for 7 days. NTX effects on CPP to oxycodone, 3 mg/kg, produced an altered dose response. The lowest doses of NTX (0.03 and 0.3 ng/kg) blocked the CPP, the middle dose (3 ng/kg) had no effect, and the highest dose (30 ng/kg) combined with oxycodone trended toward a CPP. Therefore, ultralow NTX blocked acute reward of morphine or oxycodone, in addition to blocking withdrawal-induced aversion by chronic treatment with each agonist. (Authors' comment: Low-dose NTX appears to act selectively on excitatory opioid receptors to mediate these motivational effects of interactions of the agonists.)

 Bowen et al., [87] found that mixed MOR/KOR agonists decreased cocaine i.v. intake better than selective KOR agonists in rhesus monkeys. U-50,488H and spiradoline i. p. decreased morphine and cocaine intake in rats, these effects lasting for 5-6 days in some subjects (Glick et al. [88]). The KOR effects were reversed by s.c. n-BNI. Kim et al. [89] determined that rats, when first injected with cocaine, showed an enhanced CPP to morphine and CPA to U69593. The CPA was delayed and more persistent than the CPP. Both of these effects were blocked by microinjecting MK-801 (NMDA receptor antagonist) bilaterally into the VTA, just before cocaine injection. Thus, both opioids acted upon the VTA to induce CPP or CPA. Kuzmin et al. [90] administered U-50,488H to reduce cocaine and morphine self-administration. An inverted U-shaped dose-response curve was observed for the KOR agonist, low doses enhancing self-administration, and higher doses decreasing self-administration of both morphine and cocaine.

 Negus et al. [91] described decreased cocaine self-administration by chronic administration of EKC and U-50,488H in rhesus monkeys. Cocaine self-administration interactions were also studied in rhesus monkeys by Mello and Negus [92]. Eight KOR agonists were involved, each infused over 10 days. Dose-dependent sustained reductions in cocaine self-administration were noted for EKC, Mr2033, bremazocine, U-50,488H, and enadoline, along with some decrease in food intake. Cyclazocine, PD117302, and spiradoline did not alter cocaine self-administration. EKC and U-50,488H effects were antagonized by n-BNI and NLX. Negus et al.'s [19] of testing fentanyl and spiradoline self-administration interactions was reviewed in page 4.

 Soderman and Unterwald [93] reported that cocaine CPP was attenuated by a MOR antagonist microinjected into NAcc or caudal VTA, suggesting that cocaine reward was mediated through activation of MOR receptors in either of these two brain nuclei. Additional investigation of cocaine and opioid interactions was authored by Valdez et al. [94] and Thompson et al. [95]. Valdez et al. indicated that KOR-agonist treatment in squirrel monkeys reinstated effects of cocaine, which was then attenuated by pretreating with naltrexone but not by n-BNI, suggesting a subpopulation of KOR receptors activating stress mechanisms. Thompson et al. (Shippenberg's group) described repeated dosing with U69593 to modulate DA uptake in the NAcc of rats in a manner opposite to that of cocaine, whereas acute U69593 transiently increased DA uptake. The KOR agonist also altered the activity of the DA transporter function.

 Narita et al. published a series of articles dealing with rewarding and anxiety interactions of MOR and KOR agonists in rodents (see Narita et al. [96, 97]). A chronic inflammatory state by formalin injection into rats suppressed morphine-induced reward. Pretreatment with n-BNI (KOR-1-selective antagonist) almost completely reversed this effect. Also, the morphine-induced increase in limbic forebrain DA turnover was attenuated by the inflammation, this effect being reversed by n-BNI. Therefore, inflammation may have induced a sustained activation of endogenous kappa opioid receptors in NAcc. In mice, injection of cFA or sciatic nerve ligation (SNL, neuropathic pain) produced an anxiogenic effect 4 weeks after injection or surgery. DAMGO-(MOR agonist) stimulated [^35^S]GTPgammaS binding in the amygdala was suppressed by cFA or SNL. The cFA group showed an increase in [^35^S]GTPgammaS binding in membranes of the amygdala after injection of the KOR agonist ICI199,441, suggesting an increase in receptor activation of G proteins. The authors proposed that these states of chronic pain produce anxiogenic effects and suppress MOR-agonist reward in rodents.

 Ultralow doses of NTX (1, 10, 100 pg/kg/i. v. infusion) and oxycodone interactions were examined in rats by Leri and Burns [98]. Only the lowest dose enhanced oxycodone self-administration (0.1 mg/kg/infusion), suggesting a reduced rewarding potency of the opioid agonist. During tests of reinstatement in an extinction phase, all NTX doses decreased drug seeking induced by priming injections of oxycodone (0.25 mg/kg, s.c.) or foot-shock stress. During self-administration on a progressive ratio schedule, the agonist (0.1 mg/kg/infusion) plus NTX (1 pg/kg/infusion) reached a break-point sooner compared to self-administration of oxycodone alone. Adding a NTX dose, 10 mg/kg s.c., enhanced acute stimulatory effects of the agonist (1 mg/kg, s.c.), along with increased locomotor activity by oxycodone, 7 × 1 mg/kg, s.c. So the ultralow dose NTX cotreatment augmented oxycodone locomotor activity and opioid analgesia, but reduced the agonist's rewarding potency and vulnerability to relapse. (Authors' comment: The latter two effects may have occurred through a blockade of brain excitatory opioid receptors by the ultra-low-dose NTX.)

 Funada et al. [99] blocked morphine CPP with a low dose of U-50,488H or E-2078 (KOR agonists). CPA was seen with PC using higher doses of the KOR agonists, but not with the lower doses. Pretreatment with U-50,488H or E-2078 abolished CPA due to morphine withdrawal, and this effect was reversed by pretreatment with n-BNI. U-50,488H was inactive in altering the CPP of apomorphine (DA agonist). Similar interactions of MOR and KOR agonists were reported by Tao et al. [100], Tsuji et al. [101], and Bolanos et al. [102].

 The main deterrent to the clinical application of KOR agonists as analgesics for control of chronic pain in human subjects is the disturbing side effect of dysphoria (Walker et al. [103].

 Sante et al. [104] observed a CPP in rats by microinjecting morphine into the brainstem dorsal periaqueductal gray. Microinjections of the peptide CTOP (selective MOR antagonist) or U-50,488H into dorsal periaqueductal gray induced a CPA. These results once more demonstrate mutually opposing motivational effects of brain MOR and KOR activations in specific brain nuclei (see also Koob and Le Moal [105]).

 A study of PC interactions of fentanyl (Fn) and spiradoline (Sd) in our laboratory was prompted by the hypothesis that combined s.c. MOR and KOR agonists would reduce both CPP of the MOR agonist and CPA of the KOR agonist (Rech et al. [106]). Four groups of rats, 6 in each group (A, B, C, D) were trained over five 7-day sessions in a PC procedure to saline (control group A), or dose-response levels (L = low, M = medium, and H = high) of Fn and Sd (groups B, C, and D). Shuttle responses of subjects between the two compartments were recorded by an automated recording system, avoiding potential subjective errors by an observer tabulating the subjects' movements.

 A dose-dependent CPP was formed in animals treated with fentanyl. Medium and low doses of fentanyl (0.003 and 0.006 mg/kg) were also associated with CPP, while in the same group of animals low and medium doses of Sd were capable of producing a CPA. Interestingly, a low dose of Sd reduced the CPP of a high dose of fentanyl. In addition, a medium dose of fentanyl produced a reduction in the CPA produced by a medium dose of Sd. Thus, the hypothesis in question was confirmed, that is, the KOR agonist aversion was reversed by a low dose of the MOR agonist (see also [99]). Since a low dose of KOR agonist also reduced MOR agonist reward, our results support a reciprocal interaction between drug-induced preferential and aversive motivational states. To assure that the sedative effects of both drugs were not compromising this study, we also analyzed the number of shuttles per 15 min trial for each subject as an index of locomotor activity. There was no correlation between these shuttle results and the PC data.

 Morales et al. [107] compared PC effects of morphine and U-50,488H in either a two- or three-compartment device. Morphine CPP was similar in the two instruments, but the U-50,488H CPA was better developed in the two-compartment device. They also employed an automatic recording system.

 PC was also used by Hirakawa et al. [108] in rats, to study affective responses to combined methoxamine (alpha-1-adrenergic agonist) and U69593 microinjected into RVM. Methoxamine, 0.05 mg, plus U69593, 0.178 micrograms, produced hyperalgesia in the tail-flick assay as well as CPA. Adjusted single drug doses caused CPA, no PC effect, or CPP. PC effects and spinal nociceptive reactivity showed no correlation.

 MOR- and KOR-agonist and antagonist subjective interactions in human volunteers were studied by Preston and Bigelow [109]. They administered hydromorphone (MOR agonist) and pentazocine (mixed MOR/KOR agonist) followed by NTX, 25 mg or 12.5 mg. Before NTX hydromorphone caused typical MOR effects (“liking”, calming). Pentazocine showed less intense effects of this type, along with some restlessness. The high dose of NTX blocked the effects of both agents. The 12.5 mg of NTX also blocked hydromorphone effects, but uncovered more irritability and psychotomimetic effects (typical KOR-agonist responses) as pretreatment before pentazocine. Thus, the MOR activity of pentazocine in the absence of NTX appeared to keep the drug's KOR agonist actions in check. The lower dose of NTX, selectively blocking MOR receptors, allowed for the KOR agonist influences to emerge.

## 9. Conclusions

 Combining moderate doses of a MOR agonist (fentanyl, methadone, oxymorphone, and hydromorphone) with low doses of a KOR agonist (spiradoline, enadoline, U69593, oxycodone) produced the following:

(i) additive analgesia in somatic pain assays and supra-additive analgesia in visceral pain paradigms, along with a reduction in adverse side effects such as respiratory depression, and tolerance, dependence and hyperalgesia from chronic MOR agonist treatment was attenuated by pretreatment with a KOR agonist ([14, 15, 17–19, 50, 53, 55–58], see also [67]);

(ii) analgesia of oxycodone, a KOR agonist, was superior to morphine in visceral pain states, in both animal and human subjects [41, 61–66, 75].

 Combining ultralow doses of NLX or NTX with MOR and KOR agonists resulted in

(i) enhanced analgesia, reduced tolerance, and dependence for both agonists, and decreased hyperalgesia after chronic MOR agonists [26–29, 31, 34–36, 39–44];

(ii) MOR CPP, KOR CPA, and self-administration was altered by

- reduced rewarding potency and relapse vulnerability of oxycodone [88, 90, 98, 99];
- a KOR-agonist dose too low to cause CPA (n.s. trend), which attenuated a high-dose MOR-agonist CPP and self-administration (reducing addiction liability [102, 104, 106]);
- a MOR-agonist causing modest CPP, which attenuated a high-dose KOR-agonist CPA (reducing KOR-agonist aversion), and combined medium doses of MOR and KOR agonists that resulted in mutually abolished CPP and CPA, respectively (n.s. compared to saline [106]);
- three prolonged inflammatory pain states, two with cFA [67] and [78], that abolished KOR-agonist CPA only, and the other with formalin [96], that suppressed both MOR CPP and KOR CPA.

It is tempting to speculate on the driving force for development and persistence of opposing neural MOR and KOR systems in mammalian speciation. MOR- and KOR-agonist combinations, both agents producing analgesia while provoking opposite-type side effects, may have survival value in controlling severe pain. Opposing endogenous MOR and KOR motivational/mood states in healthy subjects appear to modulate an effective balance of responses to environmental challenges [100, 103, 106, 109]. Impairments in these balances may be effectively treated by adding a low-dose antagonist (NLX, NTX) to modulate activation or suppression of inhibitory or excitatory opioid receptors.

## Abbreviations

b-FNA: Beta-funaltrexamine
CPA: Conditioned place aversion
CPP: Conditioned place preference
CRD: Colorectal distension
CWTF: Cold-water tail-flick
DAMGO: MOR agonist peptide
EKC: Ethylketocyclazocine
i.v.: Intravenous
i.c.v.: Intracerebroventricular
KOR: Kappa opioid receptor
MOR: mu opioid receptor
NAcc: Nucleus accumbens, midbrain nucleus
NLX: Naloxone
NTX: Naltrexone
PC: Place conditioning
s.c.: Subcutaneous injection
s.s.: Self-stimulation via brain electrodes
U69593: Selective KOR-1 agonist
cFA: Complete Freund's adjuvant
VTA: Ventral tegmental area
CTOP: MOR selective antagonist
DA: Dopamine
Dynorphin A: MOR agonist peptide
5-HT: 5-Hydroxytryptamine
i.p.: Intraperitoneal
i.t.: Intrathecal
KOR-1: Subtype of KOR-receptor
MR2033: KOR agonist
n-BNI: Norbinaltorphimine
n.s.: Nonsignificant
OIH: Opioid-induced hyperalgesia
RVM: Rostral-ventromedial medulla
U-50,488H: Selective KOR-1 agonist
SNL: Sciatic nerve ligation
VTA: Ventral tegmental nucleus.
